# Editorial for the Special Issue “New Strategies in Cancer Pharmacotherapy: Development of Hormonal Antineoplastic Drugs, Cytotoxic Drugs and Targeted Therapies”

**DOI:** 10.3390/ijms21114081

**Published:** 2020-06-08

**Authors:** Carlos Martínez-Campa, Carolina Alonso-González

**Affiliations:** Department of Physiology and Pharmacology, School of Medicine, University of Cantabria and Instituto de Investigación Sanitaria Valdecilla (IDIVAL), 39011 Santander, Spain

The Special Issue entitled “New Strategies in Cancer Pharmacotherapy: Development of Hormonal Antineoplastic Drugs, Cytotoxic Drugs and Targeted Therapies” was conceived with the idea of compiling information on the latest advances in the treatment of both hormone-dependent and hormone-independent cancers. The Special Issue includes both reviews and original research manuscripts addressing the efficacy of new compounds in cancer therapy. It is well known that the effectiveness of classical cytotoxic drugs commonly included in cancer treatment protocols is limited, and their use is often accompanied by severe adverse effects, which highlights the importance of the continuous search for “next-generation” compounds and/or delivery strategies. Recent developments have seen hormone analogues or antagonists, inhibitors directed against membrane receptors, monoclonal antibodies raised against cancer biomarkers, inhibitors of immune checkpoint pathways, newly designed derivatives of pre-existing molecules, or even metabolites derived from hormone-like analogues addressed in terms of their effectiveness either alone or in combination with classic cytotoxic drugs.

Kauerová et al. investigated the antitumor effects of recently designed ring-substituted 1-hydroxynaphthalene-2-carboxanilide derivatives, formulated by the extension of salicylanide structure properties. The new halogenated hydroxynaphthalene carboxamides were effective in inhibiting the proliferation of monocytic leukemia (THP-1) and breast adenocarcinoma (MCF-7) cell lines, preventing progression through G1/S transition. Moreover, one of the compounds synthesized and tested, compound 10, triggered apoptosis, suggesting that nitro-substituted hydroxynaphthalene carboxamides might represent a moiety model with promising anticancer properties [1]. Nowicki et al. focus their work on the characterization of the molecular mechanisms by which DMU-214, a metabolite of the cytotoxic resveratrol analogue DMU-212, exerts anti-proliferative and anti-migration effects in the ovarian cancer cell line SKOV-3. Whole transcriptome analysis revealed that DMU-214 triggered changes in the expression of several migration- and proliferation-related genes, providing new insights into the molecular mechanisms by which DMU-214 inhibits processes related to metastasis in ovarian cancer cells [2]. Marciano et al. have identified molecules that selectively kill cells exposed to glucose starvation. One of the identified compounds was amuvatinib, a multitargeted tyrosine kinase inhibitor. They tested the activity of 12 amuvatinib derivatives in colorectal adenocarcinoma and glioblastoma cell line cultures. One of the compounds tested, *N*-(2H-1,3-benzodioxol-5-yl)-4-{thieno[3,2-d]pyrimidin-4-yl}piperazine-1-carboxamide (compound 6), was found to be more potent than amuvatinib in a cell line-specific manner under glucose starvation, indicating that compound 6 might be a new molecule with potential anti-cancer activity. Interestingly, these compounds synergize with a vascular endothelial growth factor (VEGF) inhibitor in vivo [3]. Boschert et al. investigated the role of HGF/Met signaling in the head and neck squamous cell line with different levels of Met receptor expression. They found that Met, a crucial driver of metastasis, showed the highest expression level in a cell line derived from metastatic spread. The authors confirmed that the Met expression level is related to the amount of metabolic reprogramming, which is a factor of great relevance since targeted therapies, such as Met inhibition by tyrosine kinase inhibitors, are used in patients with advanced-stage or recurrent/metastatic disease. The study supports evidence that HGF/Met signaling maintains a central hallmark of cancer, the Warburg effect, and suggests that these metabolic changes also result in an immunosuppressive tumor microenvironment [4]. Dratkiewicz et al. generated melanoma cells resistant to vemurafenib, a B-Raf inhibitor, characterized for achieving dramatic responses initially but following this with the rapid development of resistance. Melanoma-resistant cells showed a lower proliferation rate and acquired a spindle-like shape. Increased levels of EGFR and MET were observed. Resistant cells also exhibited increased invasive abilities and elevated proteolytic activity. Moreover, combination therapy reduced their viability, indicating the efficacy of combined therapy directed against EGFR and MET with inhibitors of mutated B-Raf [5]. Aloperine, an alkaloid with a variety of pharmacological activities, has antitumor effects on human thyroid cancer cells. Yu et al. demonstrate the modulation of the autophagy mechanism in multidrug-resistant anaplastic thyroid carcinoma and multidrug-resistant papillary thyroid carcinoma cells. The underlying molecular mechanisms involve Akt/mTOR signaling pathway inhibition. The authors suggest that Akt/mTOR pathway inhibition induces both apoptosis and autophagy in human thyroid cancer cells following aloperine treatment [6]. Zareba et al. studied the anticancer properties of PAMAM G3 dendrimer generation 3 synthesized by the addition of glycidol and further modified by binding PAMAM G0 dendrimers by activation with p-nitrophenylchloroformate in human squamous cell carcinoma and human glioblastoma cells in comparison to normal skin fibroblasts. The conjugate efficiently entered the three cell lines tested and was more cytotoxic against the human squamous cell carcinoma, although it induced a strong and selective anti-proliferative effect on all cancer cell lines [7]. Zheng et al. addressed the effect of extracellular albumin in the efficacy of four (representing distinct categories) selenium (Se)-containing chemicals (SeCs) in murine myeloid leukemia and human promyelocytic leukemia cells. They found that concomitant treatment with albumin greatly decreased cytotoxicity and the cellular uptake of SeCs. They observed the formation of macromolecular conjugates between SeCs and albumin resulting in a strong inhibition of SeC uptake, possibly via albumin scavenger receptors expressed on the cell surface. Since albumin content is higher in humans than in cell cultures, the results might have clinical implications [8]. Melatonin, the pineal hormone, shows oncostatic properties and sensitizes many kinds of tumor cells to chemotherapeutic agents. In their study, González-González et al. show that melatonin also modulates the effect of docetaxel and vinorelbine not only on tumor cells but also on cells crucial in tumor microenvironments such as human mammary fibroblasts. Melatonin potentiates the stimulatory effect of docetaxel and vinorelbine on fibroblast differentiation and their inhibitory effects on aromatase activity and expression by increasing the stimulatory effect on C/EBPα and PPARγ, down-regulating antiadipogenic cytokines and COX and decreasing TNFα expression [9]. Wilms’ tumor 1 (WT1) is an intracellular oncogenic transcription factor which shows a very low expression in normal adult tissues but is overexpressed in leukemia and a variety of solid cancers. Shen et al. generated two T cell receptor mimic antibody-drug conjugates (TCRm-ADCs), ESK-MMAE and Q2L-MMAE, against WT1. Although their efficacy was not so promising (probably due to low expression), they tested a bispecific TCRm-ADC that exerted more potent tumor cytotoxicity compared with other TCRm-ADCs. The authors claim that their findings validate the feasibility of presenting intracellular peptides as the targets of ADCs, which broadens the antigen selection range of antibody-based drugs and provides new strategies for precision medicine in tumor therapy [10].

The review by Tan and Choo explores the application of pluripotent stem cells (PSCs) for the discovery of new biomarkers and generating antibodies against those biomarkers. The monoclonal antibodies generated against PSCs might have applications in cancer-targeted therapy to kill cancer cells, and conversely, monoclonal antibodies already in use in cancer might be repurposed for regenerative medicine, making it safer [11]. Grywalska et al. review the use of currently available immune checkpoint inhibitors in cervical, endometrial, and ovarian cancers. They summarize the mechanisms of action, future possibilities (vaccines), undesirable side effects, and the ongoing studies assessing combination therapies either in monotherapy or in combination with other inhibitors [12]. Finally, García Rubiño et al. review the potential of phenformin as an anticancer agent. This molecule behaves as a tumor disruptor by producing hypoglycemia due to caloric restriction. The main mechanisms involve cAMP-dependent protein kinase with energy detection and blocking of the mTOR regulatory complex. Interestingly, phenformin abrogates resistance to antiangiogenic tyrosine kinase inhibitors. The authors also review the clinical trial assays evaluating these compounds, either alone or in combination with other inhibitors, discussing current challenges and future perspectives for this biguanide [13].

Overall, the 13 contributions accepted in this Special Issue highlight the promising perspectives for analogues, targeting therapies such as monoclonal antibodies, new-generation derivatives raised from other molecules and new forms, and drug delivery in the future of cancer treatments.
